# Materia Medica

**Published:** 1889-03

**Authors:** Daniel W. Clausen

**Affiliations:** Auburn, N. Y.


### MATERIA MEDICA.

### THE PROPER SUBJECT FOR DISCUSSION IN MEDICAL-SOCIETY MEETINGS; ALSO, A FEW REMARKS ON THE DECORUM THAT OUGHT TO BE OBSERVED IN SUCH MEETINGS.

DANIEL W. CLAUSEN, M. D., *lately of Auburn, N. Y.*

(Read before The Lippe Society, Philadelphia, Pa., Tuesday evening, February, 12th, 1889.)

MR. PRESIDENT AND GENTLEMEN OF THE LIPPE SOCIETY:—I have brought with me, for the last time, a paper which had the ill-luck to be "crowded out" at our last meeting; and hope that it may not share the same misfortune on the present occasion.

Materia medica—the nature of my paper—is, I believe, generally conceded to be the soul of Homœopathy; and, in my opinion, just as the immortal soul of man gives individuality and character to his spirit, so should the soul of Homœopathy—materia medica—characterize the spirit of us who claim to be homœopathic physicians. This should be our force—our controlling spirit—when engaged individually at the bedside, and not less so when we are convened as a society for the purpose of mutual improvement in the attainments of our high and noble calling. To make our convention an opportunity for the discussion of "a paper on some disease, with the treatment," is to favor the idea of those very principles which we, as homœopathicians, strictly repudiate; for, it is one of our most forcible tenets that we "treat *patients* and *not diseases*," and it is none the less true that we cannot possibly think of "treating a disease," without in some measure favoring the materialistic idea. It is, moreover, a fact worthy of note, that, however individual a so-called "disease" may seem, we cannot treat it *per se*, without generalizing—in violation of the scientific principle of individualizing, proper to us as the professed followers of the immortal Hahnemann. To speak of "the remedies for a disease" is an absurdity; for he that would tell us of those "remedies" must, of necessity, as a homœopath, instruct us concerning *every remedy in the materia medica*, since any one remedy, never before given in a similar "disease," may be indicated in any certain case, according to those individual peculiarities appreciated by the homœopath alone. We are not to have our knowledge compressed—narrowed down—confined to a few therapeutic hints for the use of still fewer remedies; but, on the vast breadth and extent of our elaborate materia medica we are to search for "the similar" to that something in any given case of sickness, never, perhaps, before seen in a case of the kind. Verily, the unprincipled devotion of our time during the convention, to the discussion of "a paper on some disease, with the treatment," is, to say the least, an outrageous waste of time that might far better be appropriated to our improvement in that knowledge which alone can secure to us our individuality as homœopathicians—even a better acquaintance with the homœopathic materia medica. A good paper on materia medica, bringing out the genius and important features of a remedy, calls forth the most interesting and useful of all medical discussions. One member after another tells whether he has verified such or such a symptom; and, in this way, not only our knowledge, but our faith also, is built up, we become enlarged, and not narrowed down to the gill-pot measure in the treatment of disease with a few remedies. At a recent homœopathic convention, a prominent member stated that he had cured a patient of gonorrhœa with *Colchicum*, to which remedy he was guided by the peculiar symptom: *Loathing the smell of food.* Now, it is not at all likely that *Colchicum* would be included in the list of those "remedies for gonorrhœa," by the gentleman who would read us a paper on that "disease and its treatment." Let us, therefore, strive to learn something more in that department of knowledge—the homœopathic materia medica—which no living homœopath will ever live long enough to thoroughly master.

There is another point of much importance, to which I would call your attention: it is in relation to the decorum that ought to be observed in our medical meetings. We presumably convene ourselves for the purpose of mutual improvement in the learning of our profession; in other words, we come together as *students*. But while we come together as students, we do not properly do so with confusion and disorder. We have duly appointed officers who are entitled to our respect in the meeting; each individual member of us owes not only to the other members, but also to himself, a degree of respect proper to a homœopathic physician; and among the members of the assembly, individually and collectively, a due share of scholarly behavior and dignity should be observed, as becometh those who claim the title of homœopathic physicians. But, how far short of this order, when some useful and well-written paper is being read, and the reader is interrupted by the whispering and hubbub of others who thus show an utter disregard for both his material and his ability, as if they "knew it all!" This inattention and hubbub is especially offensive to the reader who is fervent in spirit and enthusiastic in his profession; and it is a forcible illustration of the truth uttered by a colored preacher on a certain occasion, at meeting, when one member of the congregation after another was called upon for "testimony." The excitement was intense, and the interruptions provoking; and the preacher exclaimed: "Brudderen, it are a fact, dat we may all *sing* togedder; but, sure as you live, we can't all *talk* togedder."

The reader or speaker, also, at a medical meeting, should not forget the etiquette and dignity proper to the occasion and to himself. Due regard for the other members, and especially for the president, beside self-respect and a desire to call the more earnest attention to the subject of this paper, should prompt him to stand upon his feet, with parliamentary observance, and pour his utterances down upon the hearers. Men like Gladstone and Disraeli could never have made Parliament feel their full power, nor could the illustrious Daniel Webster with all his eloquence have shaken the Senate as he did, had they maintained the sitting posture while speaking. I tell you, gentlemen, the power of a man is in his legs, as well as in his tongue.

These remarks concerning the decorum to be observed in medical meetings, are by no means personal; they are kindly submitted to the Society, with an ardent desire for its welfare and progress.

